# Strength and tempo of selection revealed in viral gene genealogies

**DOI:** 10.1186/1471-2148-11-220

**Published:** 2011-07-25

**Authors:** Trevor Bedford, Sarah Cobey, Mercedes Pascual

**Affiliations:** 1 Department of Ecology and Evolutionary Biology, University of Michigan, Ann Arbor, MI, USA; 2 Howard Hughes Medical Institute, University of Michigan, Ann Arbor, USA

## Abstract

**Background:**

RNA viruses evolve extremely quickly, allowing them to rapidly adapt to new environmental conditions. Viral pathogens, such as influenza virus, exploit this capacity for evolutionary change to persist within the human population despite substantial immune pressure. Understanding the process of adaptation in these viral systems is essential to our efforts to combat infectious disease.

**Results:**

Through analysis of simulated populations and sequence data from influenza A (H3N2) and measles virus, we show how phylogenetic and population genetic techniques can be used to assess the strength and temporal pattern of adaptive evolution. The action of natural selection affects the shape of the genealogical tree connecting members of an evolving population, causing deviations from the neutral expectation. The magnitude and distribution of these deviations lends insight into the historical pattern of evolution and adaptation in the viral population. We quantify the degree of ongoing adaptation in influenza and measles virus through comparison of census population size and effective population size inferred from genealogical patterns, finding a 60-fold greater deviation in influenza than in measles. We also examine the tempo of adaptation in influenza, finding evidence for both continuous and episodic change.

**Conclusions:**

Our results have important consequences for understanding the epidemiological and evolutionary dynamics of the influenza virus. Additionally, these general techniques may prove useful to assess the strength and pattern of adaptive evolution in a variety of evolving systems. They are especially powerful when assessing selection in fast-evolving populations, where temporal patterns become highly visible.

## Background

RNA viruses evolve extremely rapidly, often with mutation rates one million times greater than vertebrate species [1]. This rate of mutation allows viral populations to keep pace with rapidly changing environments. Viral pathogens, such as influenza virus, HIV, hepatitis C virus and measles virus, place a substantial burden on global human health. Often, after encountering a particular viral strain, an individual develops long-lasting immunity specific to this strain. However, in some viruses, mutations to the virus genome may result in proteins that are recognized to a lesser degree by the human immune system, leaving individuals susceptible to future infection. These mutations rapidly spread through the virus population in a process known as antigenic drift. The capacity for rapid evolutionary change allows the virus population to flourish, despite substantial immune pressure.

Understanding the process of viral adaptation is critical to our efforts to control the spread of viral pathogens. The evolution of the influenza virus has been highly studied for these reasons. Repeated epidemics of seasonal influenza infect between 10% and 20% of the human population every year, causing 250,000 to 500,000 deaths annually [2]. Mutations to the hemagglutinin (HA) and the neuraminidase (NA) genes are the primary sources of antigenic change [3]. We expect that natural selection will promote those mutations that alter the antigenic character of the virus without causing loss of function of the HA and NA proteins. Previous studies have focused on rates of nucleotide and protein change, examining the ratio of the rates of nonsynonymous change to synonymous change (*dN*/*dS*) in the HA genealogical tree [4-6]. Deleterious mutations are weeded out from the population, resulting in an overabundance of deleterious mutations on the side branches of the tree, while advantageous mutations spread through the entire population, resulting in an overabundance of advantageous mutations on the trunk of the tree. These studies have shown that epitope sites evolve extremely rapidly on the trunk of the genealogical tree, indicative of the presence of adaptive evolution [5,6].

Although it is well accepted that adaptive evolution occurs in influenza, there has been considerable debate as to the tempo of this adaptation. Understanding the relative importance of continuous vs. episodic change appears critical to an adequate explanation of influenza epidemiological dynamics. Some epidemiological models have assumed continuous antigenic change [7-9], while others have assumed episodic antigenic change [10]. These models have sought to understand the general patterns in genealogical trees of specific influenza A subtypes and in particular, the mechanisms that limit the genetic diversity of the virus despite its high transmissibility and mutation rate. Alternative hypotheses have resulted from these theoretical efforts, emphasizing on one hand selective sweeps and episodic antigenic change [10], and on the other, continuous selection and short-lived strain-transcending immunity [7]. In empirical studies of patterns of nucleotide and protein change, some authors have argued that adaptive change is continuous [11,12], while others have argued that adaptive change is discontinuous, occurring in an episodic fashion [6]. Generally, much of this difficulty in analysis arises from the complex mapping of genotype to antigenic phenotype in influenza; antigenic character may change substantially without a corresponding genetic leap [13]. Because of the complexity of the genotype-phenotype map, it will necessarily be difficult to directly extrapolate the presence of sequence changes to an inference of antigenic innovation. Instead, we propose using temporally tagged genetic data to examine the large-scale dynamics of a viral population, searching for traces left by adaptive evolution in the shape of genealogical trees. This analysis uses phylogenetic and population genetic techniques to characterize the strength and the timing of adaptive change using sequence data.

## Results and Discussion

### Effective population size

**Figure 1:**
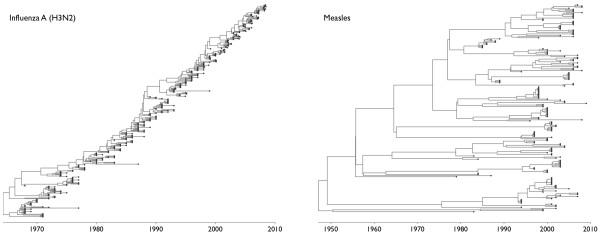
**Genealogical trees derived from hemagglutinin sequences of influenza A (H3N2) and measles virus**. The influenza tree, sampled between 1968 and 2008, appears long and spindly with a distinct lack of deep branches. It only takes a few years for contemporaneous strains to find a common ancestor. The measles tree, sampled between 1979 and 2009, looks very different, harboring many deep branches. It takes approximately 50 years for contemporaneous measles strains to find a common ancestor.

The genealogical trees of the hemagglutinin genes of human influenza A (H3N2) and human measles virus appear immediately distinct, with influenza exhibiting a ladder-like tree with a long trunk and very short side branches and measles presenting a bushier tree with deeper branchings (Figure 1). In influenza, the trunk branch corresponds to the progenitor lineage; mutations that occur along the trunk are eventually *fixed*, persisting until 'over-written' by subsequent mutations. In contrast, mutations that appear on side branches are eventually *lost *from the population. We expect that a similar progenitor lineage exists in measles, but because the population turns over much more slowly, we do not have enough temporal resolution to identify this progenitor lineage.

The competitive mechanisms by which the viral populations replace themselves have important repercussions for their genealogical trees. It is well known that influenza undergoes extensive antigenic drift, allowing a single individual to be repeatedly infected with successive strains of influenza differing in antigenic character [2]. The influenza vaccine strain must be continually updated to remain effective against infection. In contrast, primary infection by measles confers strong lifelong immunity, allowing vaccination to remain effective through time [14]. In influenza, antigenic drift creates competition among strains, resulting in the emergence of selective forces. Here, selection that promotes antigenically novel strains results in rapid population turnover; in measles, the immune response is equally potent across strains, resulting in neutral evolutionary dynamics and slower strain turnover (Figure 1). The interface between phylogenetic patterns and ecological dynamics has been referred to as 'phylodynamics' [15]. Previous work on phylodynamic patterns has focused on the obvious visible differences between the genealogical trees of influenza and of measles [15]. By calculating the effective size of each viral population, we attempt to quantify and explain the differences between the sparse tree of influenza and the prolific tree of measles.

**Figure 2:**
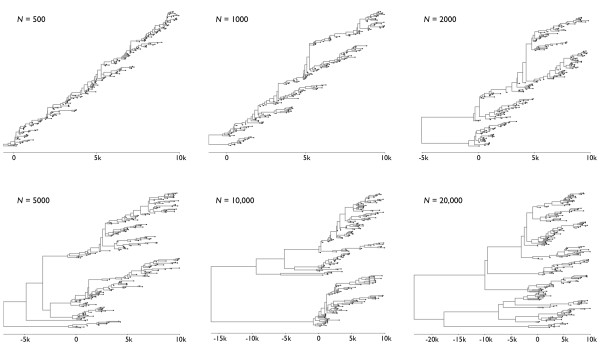
**Genealogical trees derived from simulated sequences at varying population sizes**. In each case, sequence evolution was simulated using a neutral Wright-Fisher model, and approximately 200 samples were taken over the course of 10,000 generations (sampled at a Poisson rate of 0.02 per generation). In each frame, the *x*-axis is shown as intervals of 5000 generations, so that the temporal scale increases with *N*. As population size *N *increases, the time it takes for two lineages to coalesce increases proportionally, so that larger populations show a deeper most recent common ancestor. Population dynamics result in rapid coalescence when there are many contemporaneous lineages and slower coalescence when there are few, a detail especially apparent at larger population sizes.

Effective population size *N_e _*demarks the timescale at which population turnover occurs. On average, we expect 2*N_e _*generations for a new mutation to fix in a haploid population, and we expect *N*_e _generations for two randomly chosen individuals to find a common ancestor, a process known as coalescence [16]. The overall appearance of a genealogical tree will depend upon the rate at which lineages coalesce with one another, determined by *N_e_*, along with the degree of temporal spacing among samples. If coalescence is fast relative to the rate at which sampling occurs, then the resulting genealogy will appear ladder-like and spindly, while if coalescence is slow relative to sampling patterns, then the resulting genealogy will appear dense and bushy (Figure 2). The coalescent timescale exhibited by natural populations will depend on both effective population size *N_e _*and on generation time *τ*, often measured in years per generation. Thus, a population of *N_e _*= 100 effective individuals with *τ *= 0.01 years per generation will have a timescale of coalescence of *N_e_τ *= 1 year.

From sequence data alone, it is impossible to independently identify *N_e _*and *τ*; only their product *N_e_τ *can be estimated. We fit a phylogenetic model incorporating the coalescent process to sequence data to estimate genealogical trees and coalescent parameters for influenza and measles virus (Figure 1). In this case, we estimate *N*_e_*τ *for HA of influenza A (H3N2) at 7.2 years (95% credible interval 6.2-8.4) and *N*_e_*τ *for measles at 124.6 years (100.4-153.9). These estimates give quantification to the visual differences apparent in genealogical trees. We arrive at independent estimates of *τ *via experimental and epidemiological data on the duration of infection, which we take as 5 days for influenza (*τ *= 0.014 years per generation) [17] and 11 days for measles (*τ *= 0.030 years per generation) [14]. Our basic results do not depend on these exact estimates. Dividing by *τ *yields estimates of *N_e _*= 526 (451-611) for influenza and *N_e _*= 4135 (3332-5107) for measles.

In the absence of complicating factors such as selection, spatial population structure or fluctuating population size, effective population size *N_e _*is expected to equal consensus population size *N*. We arrive at very rough estimates of the census sizes of influenza and measles via overall incidence. There are approximately 5 × 10^8 ^cases per year of influenza [18], which spread out over 5 day intervals gives an average of *N *= 7 × 10^6 ^cases per generation. There are about 3 × 10^7 ^cases per year of measles [19] or *N *= 9 × 10^5 ^cases per generation. Here, the difference between the census population size and the effective population size (*N*/*N_e_*) of influenza is enormous, approximately 13,000, while the difference in measles is much smaller, approximately 220.

The observed differences between *N_e _*and *N *in influenza and measles may be due to multiple causes. Anything that skews reproductive output, so that a small number of infections contribute disproportionately to the genetic legacy of viral population, will inflate *N*/*N_e_*. Factors could include spatial population structure or other variation in host contact patterns. However, it is a well known population genetic finding that selection, especially positive selection, reduces the effective size of an evolving population [20]. This is why selective sweeps are identified in *Drosophila *and in humans by finding regions of the genome with low diversity, indicative of decreased effective population size [20]. We may expect most of the discrepancy between influenza and measles to be due to the effects of natural selection, as both infections represent acute viral infections displaying strong seasonal epidemics. Thus, without looking at rates of evolution, we can infer that levels of selection are substantially greater in influenza than in measles. If we were to assume complete equivalence of epidemiological factors such as seasonality and spatial structure, then we would estimate levels of selection in influenza as 13000/220 = 59 times greater than in measles. This level of selection is an average, and it may arise from frequent fixation of weakly adaptive mutations or from rare mutations of large adaptive impact. We investigate a more detailed dataset in an attempt to distinguish between these hypotheses.

### Turnover of simulated sequences

**Figure 3:**
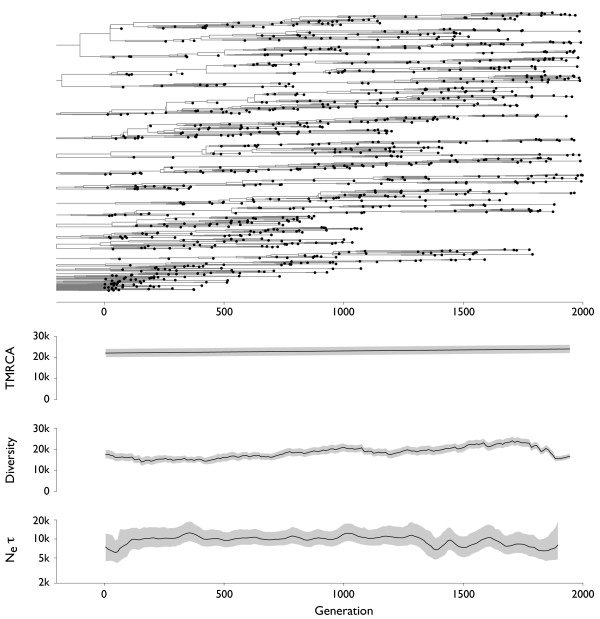
**Genealogical tree and skyline plots of coalescent statistics for sequences simulated under selective neutrality**. There were 10,000 individuals in the simulation. The tree shown represents the maximum posterior tree sampled over the course of the MCMC analysis of the simulated data. For coalescent statistics, TMRCA, diversity and *N_e_τ*, solid lines represent mean values and gray outlines represent 95% credible intervals across MCMC replicates. TMRCA, diversity and *N_e_τ *are measured in units of generations.

**Figure 4:**
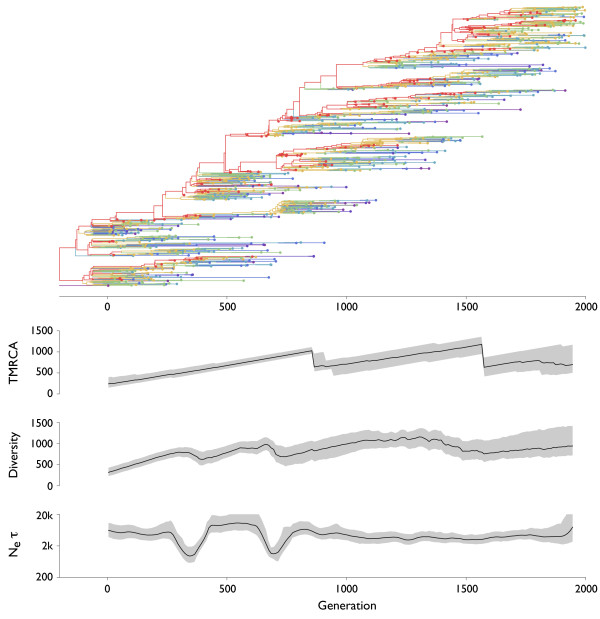
**Genealogical tree and skyline plots of coalescent statistics for sequences simulated under purifying selection**. There were 10,000 individuals in the simulation, and a mutation rate *μ *of 10^-5 ^per site per individual per generation with each mutation having a 99% probability of being deleterious with a selective disadvantage of 0.01. The tree shown represents the maximum posterior tree sampled over the course of the MCMC analysis of the simulated data. Colors move from red to purple as fitness decreases. For coalescent statistics, TMRCA, diversity and *N_e_τ*, solid lines represent mean values and gray outlines represent 95% credible intervals across MCMC replicates. TMRCA, diversity and *N_e_τ *are measured in units of generations.

**Figure 5:**
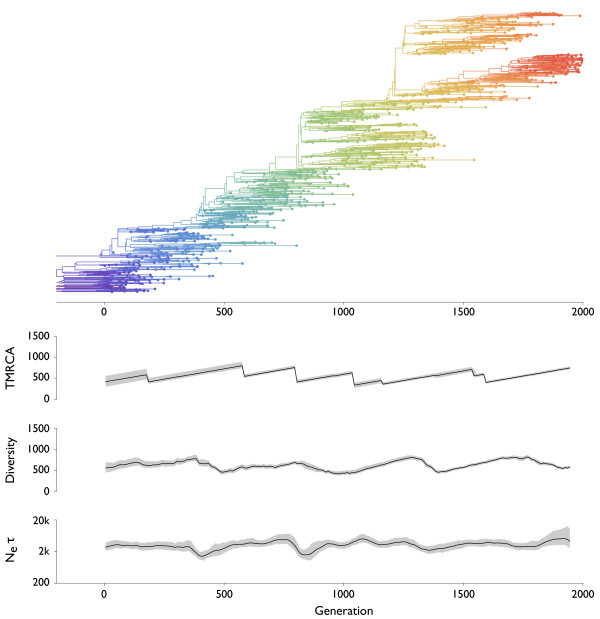
**Genealogical tree and skyline plots of coalescent statistics for sequences simulated under constant positive selection**. There were 10,000 individuals in the simulation, and an advantageous mutation rate *μ_a _*of 0.002 per individual per generation with each mutation harboring a selective advantage *s *of 0.01. The tree shown represents the maximum posterior tree sampled over the course of the MCMC analysis of the simulated data. Colors move from purple to red as fitness increases. For coalescent statistics, TMRCA, diversity and *N_e_τ*, solid lines represent mean values and gray outlines represent 95% credible intervals across MCMC replicates. TMRCA, diversity and *N_e_τ *are measured in units of generations.

**Figure 6:**
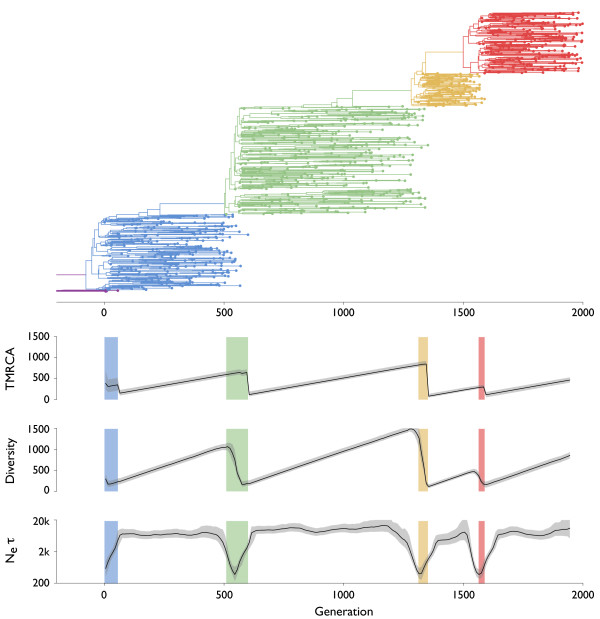
**Genealogical tree and skyline plots of coalescent statistics for sequences simulated under episodic positive selection**. There were 10,000 individuals in the simulation, and an advantageous mutation rate *μ_a _*of 8 × 10^-7 ^per individual per generation with each mutation harboring a selective advantage *s *of 0.1. The tree shown represents the maximum posterior tree sampled over the course of the MCMC analysis of the simulated data. Colors move from purple to red as fitness increases. For coalescent statistics, TMRCA, diversity and *N_e_τ*, solid lines represent mean values and gray outlines represent 95% credible intervals across MCMC replicates. TMRCA, diversity and *N_e_τ *are measured in units of generations.

To better describe the effects of modes of selection on tree topology, we undertook a simulation-based study, in which models of neutral evolution (Figure 3), purifying selection (Figure 4), constant positive selection (Figure 5) and episodic positive selection (Figure 6) were compared. In each case, an evolving population of 10,000 sequences was simulated according to a Wright-Fisher model with non-overlapping generations. Each sequence consisted of 2000 bases and mutated at a rate of 10^-5 ^per site per generation. In the neutral simulation, each sequence was assigned equivalent fitness. In the simulation of purifying selection, each mutation had a 99% chance to be deleterious, and deleterious mutants had a selective disadvantage of 0.01 relative to their parent's fitness. In the simulation of constant positive selection, each mutation had a 10% chance to be advantageous, and advantageous mutants had a selective advantage *s *of 0.01 relative to their parent's fitness. In the simulation of episodic positive selection, each mutation had a 0.004% chance to yield a selective advantage of *s *= 0.1. These parameters were chosen so that the overall genetic diversity the population was similar in all three cases with selection. In the cases of constant positive selection and episodic positive selection, it took an average of ~5000 generations for the population to double in fitness. The population-scaled rate of advantageous mutation *Nμ_a _*was 20 per generation for constant selection and 0.008 per generation for episodic selection. The population-scaled selective advantage *Ns *was 100 for constant selection and 1000 for episodic selection.

In each simulation, sequences were sampled over the course of 2000 generations at a rate of one sample per 2 generations to yield approximately 1000 samples in total. A Bayesian coalescent approach was then used to estimate the gene genealogies connecting these sequences (Figure 3, 4, 5, 6). It is immediately obvious from these results that selection constrains tree topology, resulting in more narrow ladder-like trees in each case of selection relative to the case of neutrality. Both positive and negative selection constrain both genetic diversity and scaled effective population size *N_e_τ*. Under purifying selection, the original phenotype remains throughout the simulation (red lineage in Figure 4), but mutations to this lineage often result in less fit phenotypes. These lineages are quickly purged from the population (Figure 4). In this case, the mean fitness of the population remains roughly constant through time, while in both cases of positive selection fitness increases through time (Figure 5, 6). There are further differences between the case of constant positive selection and the case of episodic positive selection. In the case of constant positive selection (Figure 5), the tree appears fairly uniform, although there are multiple cases where relatively major lineages die out. Here, the effects of clonal interference [21,22] are apparent. All major lineages continually accumulate beneficial mutations. However, because there is no recombination, these mutations cannot be combined onto the same genetic background, and thus, they are in competition with one another. The tree shows that lineages that stochastically accrue more beneficial mutations tend to outcompete less fortunate lineages. In the case of episodic positive selection (Figure 6), we see that mutation events result in a rapid transition of the population to the new allelic state. This rapid transition can be seen as a sudden crash in genetic diversity, corresponding to the loss of lineages (side branches) bearing the ancestral allele. As opposed to what is seen with constant selection, the disappearance of these lineages is synchronous, e.g. multiple green lineages simultaneously disappear when yellow emerges at around generation 1300.

A gene tree represents a complex, high-dimensional description of the genealogical relation-ships among a sample of sequences, and cannot be completely summarized by a single statistic. Instead, summary statistics can be used to probe various aspects of tree topology. Common summary statistics applicable in this context include the time to the most common ancestor (TMRCA) of the sample and the mean pairwise diversity of the sample, which is the average branch length distance between pairs of tips on the tree. Here, both TMRCA and diversity are measured in the same units (generations) as the branches in the tree. Additionally, the rate of coalescence can be directly measured from a genealogy by weighting the number of coalescent events observed by the total opportunity for coalescence [23,24]. The inverse of this rate of coalescence yields an estimate of the scaled effective population size *N_e_τ *[16]. For each of these three statistics, we analyzed the posterior sample of genealogical trees to get a mean estimate and a 95% credible interval at multiple points in time. In the cases of TMRCA and diversity, at each point in time we took a 'slice' of contemporaneous lineages and traced their ancestry backward in time until arriving at a common ancestor. This effectively turns a tree with temporally heterogeneous tips into a tree with all tips at the same point in time. Temporal 'slices' were taken every 10 generations. To calculate *N_e_τ*, we analyzed the rate of coalescence in sliding windows of 50 generations in width, moving the center of the window 10 generations in each step. For comparison, the 'Bayesian skyline plot' (BSP) [23,24] popularized by the software package BEAST [25] shows the same information as our plots of *N_e_τ *through time in Figures 3, 4, 5, 6. If time is measured in generations then *N_e_τ *will be measured in generations, and if time is measured in years then *N_e_τ *will be measured in years. However, in the literature the quantity measured by the BSP is sometimes referred to as 'relative genetic diversity' [26]. We disagree with this terminology and prefer to use 'diversity' to describe the sequence or temporal distance between lineages, as is standard in the population genetics literature [20]. At equilibrium, an increase in *N_e_τ *won't result in an immediate increase in diversity; instead, diversity will slowly increase until an equilibrium compatible with the new value of *N_e_τ *is reached.

Under the neutral coalescent, TMRCA and diversity are expected to equal 2*N *= 20,000 generations and effective population size is expected to be equivalent in generations to the census population size of 10,000 individuals. Coalescent statistics for the neutral case are in line with these expectations (Table 1 Figure 3). As predicted, measures of TMRCA, diversity and to a lesser extent *N_e_τ *are lower in cases with selection (Table 1 Figure 5, 6). Here, the ancestry of the population is funneled through a small number of superior individuals, resulting in a decrease in genetic diversity. We observe that TMRCA, diversity and *N_e_τ *remain consistently low under constant negative and positive selection (Figure 4, 5). In the case of constant positive selection, there is some evidence of low amplitude cycles in levels of diversity (Figure 5). However, under episodic selection, we observe large fluctuations in TMRCA, diversity and *N_e_τ *(Figure 6). During periods of selective neutrality, *N_e_τ *coincides with the expected 10,000 generations and TMRCA and diversity gradually increase over time. This increase would continue until the neutral expectation of 2*N *generations were reached. However, when a selectively advantageous mutation appears, it very quickly sweeps through the population. We observe a crash in diversity coinciding with the appearance of the newly emerged, advantageous mutation and a drop in TMRCA after the sweep is complete. During the course of the selective sweep, *N_e_τ *drops precipitously, but immediately recovers after the sweep is over.

**Table 1:** Means and 95% range across 2000 generations for various coalescent statistics on samples of genealogical trees generated from simulated sequences.

	TMRCA^1^	Diversity^1^	*N_e_τ*^1^
Neutrality	23.09 (22.16-24.02)	18.38 (14.48-23.27)	10.01 (6.50-12.26)
Purifying selection	0.74 (0.27-1.14)	0.85 (0.38-1.14)	3.96 (1.12-10.35)
Constant positive selection	0.57 (0.37-0.77)	0.63 (0.43-0.81)	3.25 (1.57-5.00)
Episodic positive selection	0.35 (0.12-0.82)	0.58 (0.17-1.43)	7.19 (0.41-12.54)

^1 ^Measured in units of 10^3 ^generations.

We propose to use temporal patterns of diversity and scaled effective population size as tools to judge patterns of selection in viral genealogies. Both positive and negative selection result in a reduction of diversity. If episodic selection predominates, then we should observe slow growth and occasional rapid decline in diversity coupled with a transient drop in *N_e_τ*, while if constant selection predominates, then we should observe much flatter patterns of diversity and *N_e_τ *through time. Here, our use of TMRCA and diversity, complements and extends previous analyses relying on 'Bayesian skyline plots' of *N_e_τ*. For a sweep to be found with *N_e_τ*, it requires catching the sweep in action, while a sweep's effects on diversity are more long-lasting. Our use of genealogical summary statistics is similar to that of Drummond et al. [27]. We recognize that our application of a neutral coalescent prior in the phylogenetic inference process is non-ideal. Phylogenetic methods that directly incorporate the actions of selection into their models of tree topology would be preferred. Currently, there are theoretical efforts in directly modeling selection in a coalescent framework, e.g. the ancestral selection graph [28], but these approaches lack suitable implementations for the viral genealogies presented here. In our approach, were use a flexible Bayesian model that gives important insight into the effects of selection through the parameter *N_e_τ *and through genealogy shape. Here, the data will push the final genealogical inference away from the neutral coalescent expectation. As such, we can identify the presence of selection, even if a parameter such an selective effect *s *is lacking from the phylogenetic model.

### Interaction of selection and population size

**Figure 7:**
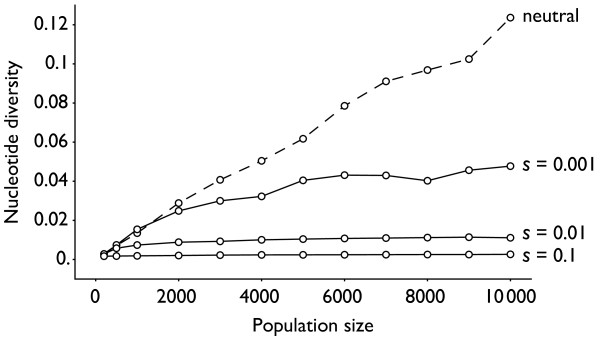
**Relationship between population size *N *and genetic diversity at varying intensities of selection**. Nucleotide diversity represents mean pairwise proportion of sequence differences between random individuals in the population. Diversity estimates are taken from 10^5 ^generations across 3 replicate simulations. Neutral dynamics are shown as a dashed line. Selective dynamics are shown as solid lines. In each case, 1% of mutations are selectively advantageous, with a selective advantage *s *of 0.001, 0.01 or 0.1.

It is clear that viral genealogies should be affected by changes in pathogen population size (as described by prevalence patterns), as well as by selective dynamics. In the absence of selection, we expect a general correspondence between increases in prevalence and increases in *N_e_τ*. In this case, *N_e _*should be strickly equivalent to prevalence. However, *τ *will vary over the course of an epidemic as susceptibles become depleted [29]. During the initial upswing, *τ *will be inversely proportional to contact rate, while at equilibrium, *τ *will be inversely proportional to rate of recovery. Other authors have extensively demonstrated this correspondence in simulation studies [24,26]. However, with selection in play, it is not immediately clear what relationship we should expect between prevalence and *N_e_τ*. We investigated this relationship using further simulations. As before, an evolving population of sequences was simulated according to Wright-Fisher dynamics. Mutations occurred at a rate of 10^-5 ^per site per generation, and 1% of mutations were advantageous. We ran a number of simulations to ascertain the nucleotide diversity, measured as the per-site proportion of nucleotide differences between random pairs of sequences in the population, as a function of the population size *N *and the selective coefficient of advantageous mutations *s*. In each case, diversity was estimated as the mean of 10^5 ^generations of simulation after 10^5 ^generations of burn-in across three replicate simulations. The results show that with strong selection, genetic diversity is effectively divorced from increases in population size (Figure 7). Under neutrality, genetic diversity increases linearly with population size, but with selection, initial increases in genetic diversity occur at low population sizes, but diversity plateaus at higher population sizes. This suggests that, in cases of strong selection, the ancestry of sequences is determined by selective dynamics and the rapid fixation of advantageous alleles, rather than by the forces of genetic drift as determined by population size [20].

Measles and influenza show vastly different genealogical patterns (Figure 1); it appears that strains of influenza undergo strong selective pressures based upon their antigenic phenotypes, while strains of measles are antigenically similar and exhibit neutral dynamics. Our results suggest that the genealogical patterns of measles are strongly impacted by epidemiological patterns, where increases in prevalence more-or-less directly map to increases in *N_e_τ*, while the genealogical patterns of influenza are primarily driven by selective dynamics. Still, it is clear that prevalence impacts the genealogical patterns of influenza. Looking just within the USA and within New Zealand influenza samples, Rambaut et al. [26] show that *N_e_τ *drops during the summer months and peaks during the winter months, in accordance with seasonal fluctuations.

### Selective dynamics of influenza A (H3N2)

**Figure 8:**
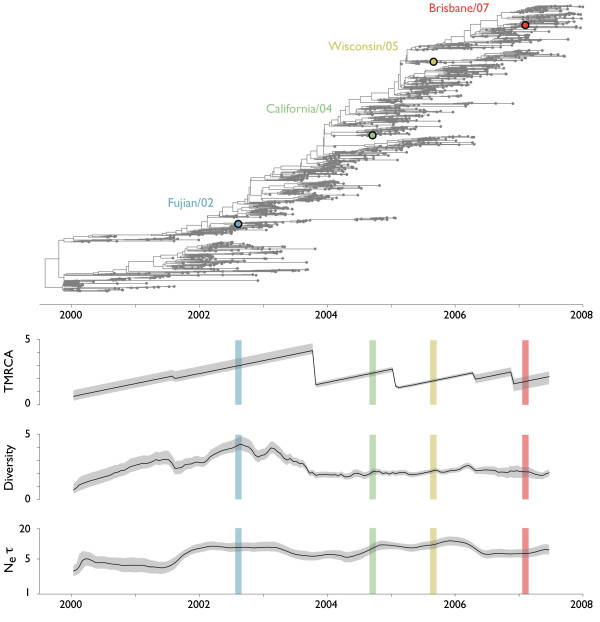
**Genealogical tree and skyline plots of coalescent statistics for 1270 world-wide samples of HA sequences of influenza A (H3N2)**. The tree shown represents the maximum posterior tree sampled over the course of the MCMC analysis. Vaccine strains are shown on the tree as colored points. For coalescent statistics, TMRCA, diversity and *N_e_τ*, solid lines represent mean values and gray outlines represent 95% credible intervals across MCMC replicates. TMRCA, diversity and *N_e_τ *are measured in units of years.

A more detailed look at the recent evolutionary history of the hemagglutinin gene of influenza A (H3N2) further reveals the impact of selection on tree topology. World-wide samples of influenza A (H3N2) taken between 2000 and 2008 show a pattern of rapid population turnover, with most side-branches persisting for 2-3 years (Figure 8). The overall estimate of *N_e_τ *for these 1270 sequences is very similar to the estimate of *N_e_τ *from many fewer sequences sampled over a longer time period (6.7 years and 7.2 years respectively). As discussed previously, the overall scale of the influenza tree as seen in *N_e_τ *is indicative of selection, however the shape of the influenza genealogy further suggests its pervasive influence. If neutral dynamics are occurring, then we expect to find TMRCA and diversity to be 2× greater than *N_e_τ*. This pattern is supported by our neutral simulation (Table 1). However, if selection is present then this ratio will be distorted, as it is the case of all simulations involving selection (Table 1). In influenza, we find that the ratio of TMRCA to *N_e_τ *is 0.36 (0.12-0.80) and the ratio of diversity to *N_e_τ *is 0.39 (0.16-0.95) (Table 2). We are confident that most of these patterns are driven by positive, rather than negative, selection. Measles virus undergoes purifying selection to maintain its function [30], yet its genealogy appears largely congruent with neutral dynamics. Other genetic studies of influenza have shown abundant evidence for adaptive evolution of the HA1 domain, the primary target of the human immune response [5,6,31].

**Table 2:** Means and 95% range from 2000 to 2007.5 for various coalescent statistics on samples of genealogical trees generated from influenza A (H3N2) sequences.

	TMRCA^1^	Diversity^1^	*N_e_τ*^1^
China & SE Asia	1.75 (0.48-2.81)	1.78 (0.70-2.73)	3.30 (2.17-5.18)
Worldwide	2.10 (0.79-4.00)	2.20 (1.21-4.07)	6.72 (3.36-11.18)

^1^Measured in years.

Interesting temporal dynamics are apparent in the turnover of the influenza population. From 1999 to 2004, two major lineages of influenza steadily diversified their HA genes. Coexistence of these lineages leads to a steady increase in TMRCA and diversity during this time. TMRCA increases until one of the lineages disappears just before 2004, while diversity increases until mid-2002, at which point it begins to decline, reaching a minimum in 2004. After 2004, the dynamics of the influenza population appear distinct from the dynamics of the earlier period. From 2004 to 2008, side-branches continually emerge and die off, so that TMRCA and diversity remain at a fairly low and constant level. Given previous simulation results, a reasonable hypothesis for the selective dynamics from 2000 to 2008 would be a period of somewhat relaxed selection from 2000 to mid-2002 allowing diversity to accumulate, a selective event in 2002 that causes diversity to decline and TMRCA to eventually drop, and a period of continuous selection from 2004 to 2008 that keeps diversity low. However, even during the 2000 to 2003 period, *N_e_τ *remains far below its neutral expectation, suggesting that some degree of selection is always present. The slight increase in *N_e_τ *after 2002 is likely due to sampling effects, before 2002 samples are restricted to China, Oceania and the USA, while after 2002, sampling is more global. During this time period, there were no major shifts in influenza prevalence which could explain these genealogical patterns.

The preceding description, gathered through genetic analysis alone, qualitatively agrees with known patterns of antigenic evolution in the HA gene. From 2000 to 2002, the virus population remained in the Sydney/97 antigenic cluster and evolved very little in antigenic phenotype, although continued to evolve in genotype [13]. This pattern is corroborated by our finding of increasing diversity during this time period; antigenic change provides the fuel for selection among strains, and so without antigenic evolution we expect a build up of genetic diversity. In 2002, the Fujian/02 strain emerged from the tropics and effected a large jump in antigenic phenotype [13]. This antigenically novel variant rapidly spread through the global influenza population, and through this rapid spread displaced genetic diversity during the course of 2002-2003. The period from 2004 to 2007 saw a large amount of antigenic change, first with the California/04 transition and later with more continuous, but rapid antigenic change, necessitating vaccine updates for Wisconsin/05 and Brisbane/07 [32]. This pattern of sustained antigenic evolution agrees well with our observation of continuously low levels of genetic diversity.

The observed consistently low levels of diversity from 2004 to 2008 are indicative of continuous selection on the global influenza population. However, we caution that this result is only applicable at a fairly coarse temporal resolution. From simulation, it can be seen that if a selective events occur sufficiently close together, there will not be time for diversity to recover and thus the second selective event will remain to a large extent hidden (Figure 6). It is thus possible that our observations of continuously low diversity from 2004 to 2008 can be explained by relatively few selective events, perhaps close to 4 in the 4 year period. It is also, of course, possible for there to have been more than 4 such events during this time.

**Figure 9:**
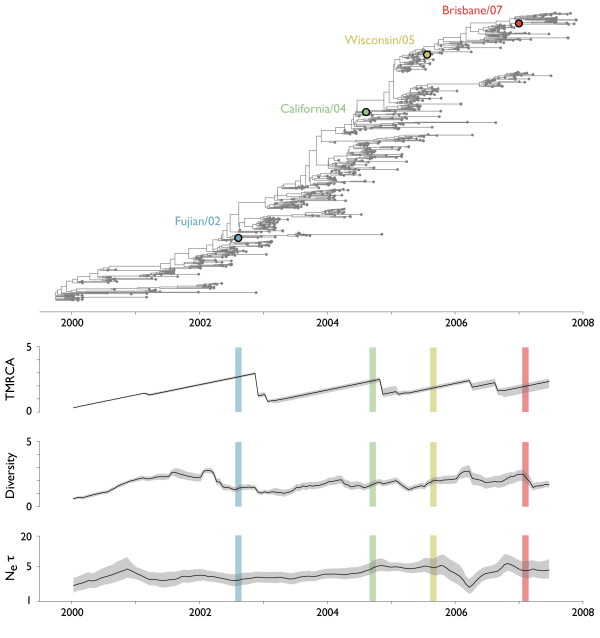
**Genealogical tree and skyline plots of coalescent statistics for 624 China and Southeast Asia samples of HA sequences of influenza A (H3N2)**. The tree shown represents the maximum posterior tree sampled over the course of the MCMC analysis. Vaccine strains are shown on the tree as colored points and included regardless of geographic origin. For coalescent statistics, TMRCA, diversity and *N_e_τ*, solid lines represent mean values and gray outlines represent 95% credible intervals across MCMC replicates. TMRCA, diversity and *N_e_τ *are measured in units of years.

Patterns of diversity may be influenced by the spatial structure of the global virus population. Indeed, previous studies have shown that metapopulation dynamics promote persistence and shape genealogical patterns [32,33]. To gain insight into the confounding influence of spatial structure on our results, we repeated the analysis of diversity patterns on samples from within China and Southeast Asia (Table 2 Figure 9). As expected, overall levels of diversity and *N_e_τ *are lower for the regional influenza population compared to the global population, although the impact was stronger on *N_e_τ *than diversity (Table 2). We see a similar temporal pattern of growth and decline in genetic diversity for the regional China and Southeast Asia samples (Figure 9). Diversity grows from 2000 to 2002, declines from 2002 to 2003 and increases slowly from 2003 to 2007; there is more variation in post-2004 diversity here than seen in the global analysis.

The Fujian/02 antigenic variant was first discovered in southeastern China. Consistent with this geographic origin, diversity within China and Southeast Asia begins to decline in early 2002, as opposed to the global decline observed in mid-2002. The vaccine strain Fujian/02 was not sampled until August 2002. Our analysis suggests that the original antigenic variant that effected the cluster transition first appeared significantly earlier. Generally, it may be possible that the relatively slow decline in diversity observed during 2002-2003 may be in part due to a spatially asynchronous global influenza population. In this case, a selective sweep will necessarily be constrained by waiting for migration events to spread the novel variant throughout the world [33].

Thus, it appears that there is a broad correspondence between patterns of antigenic change and patterns of genetic diversity. It is generally thought that the HA1 domain of the HA protein experiences the strongest degree of immune pressure because of its high concentration of epitope-associated sites [5]. Most antigenic changes in this period appear largely due to amino acid substitutions in epitopes A and B and the receptor binding domain of HA [34]. However, it has recently been suggested that the evolution of antigenicity and receptor specificity are intimately linked, implying that selection on one will lead to changes in the other [35]. This picture is further complicated by variation in the number of potential N-linked glycosylation sites (PNGS) on the surface of HA over this period. Glycans attached to PNGS have been shown to play a role in antibody escape, the strength of receptor binding, oligomerization, sorting, and transport [36,37]. Because the addition of PNGS to H3 HA appears to increase viral clearance by components of innate immunity, the fact that there has been an overall increase in the number of PNGS on H3N2 HA since 1968 suggests its other functions have greater selective importance [37,38]. It is feasible that amino acid substitutions in the epitopes in conjunction with the addition and removal of PNGS result in frequent, incremental changes in strain fitness and antigenicity.

The effects of other genes on selection in H3 HA are probably secondary. Analysis of sequences collected in New York and New Zealand from 1995 to 2008 showed that the HA gene had consistently the smallest TMRCA [26], indicating it is under strong selection and thus the least likely hitchhiker. The gene whose function is most closely linked to that of HA is NA, which must compensate for changes in the receptor binding affinity of HA. After HA, NA is under the strongest positive selection and is an antibody target [31]. In Denmark, amino acid substitutions in N2 NA epitopes were observed in every season from 1999-2000 to 2005-2006, in addition to substitutions in CTL epitopes in other genes [39]. It is hard to gauge the extent to which genome segments evolve independently. On the one hand, reassortment rates are high, with fourteen separate events estimated from analysis of relatively small populations over four seasons [40]. On the other hand, subsets of the genome significantly differ in the amount of phylogenetic history they share with one another [26], and their genes have uniquely different functions and constraints.

## Conclusions

Multiple epidemiological models have been put forward in an attempt to explain the observed patterns of diversity in the influenza virus [7-10]. In standard population genetic models, selection based on Malthusian fitness, or *R*_0 _in the case of pathogens, will always result in decreased genetic diversity and effective population size. However, selection for antigenic novelty may result in extended or deep bifurcations of the pathogen population, where a sub-set of the population moves in one antigenic (and genetic) direction and another subset moves in another antigenic direction. Typically, this bifurcation leads to stable coexistence of multiple lineages, despite the presence of strong immune pressure on the viral population. Any epidemiological explanation of influenza A dynamics must simultaneously generate strong immune pressure, while preventing prolonged bifurcation of the influenza population.

In this study, we have shown that observed patterns of population turnover corroborate patterns of antigenic change from 2000 to 2008. In particular, an epidemiological model of influenza must be able to generate periods of relative antigenic stasis and increasing genetic diversity, as observed from 2000 to 2002, but also periods of sustained antigenic change and consistently low genetic diversity, as observed from 2004 to 2008. The results here provide partial support for the idea that periodic selective sweeps can play a role in the low diversity of flu (as evidenced by the Fujian/02 transition) [10], but they also suggest that these sweeps are not necessary and cannot be the only mechanism at play (given the diversity results for 2004-2008). Thus, even if transitions between antigenic clusters play a strong role in shaping longer-term influenza dynamics [10,13], it appears another force must at times be responsible for maintaining low diversity in HA. In the limiting case of epochal evolution, we would expect to see very large values of *N_e_τ *during periods of antigenic stasis indicative of selective neutrality. However, results for *N_e_τ *between 2000 and 2002 strongly suggest that selection is continuously operating in influenza. The strength of selection appears to vary somewhat over time, but its presence is seen throughout the study period.

Interestingly, none of the existing epidemiological models that explicitly incorporate both evolution in sequence space and selection through immunity and cross-immunity in phenotypic space appear able to explain the two different periods observed in the data. A model with short-lived transcending immunity [7] would be compatible with the latter but not the former period; while the opposite would be the case for a model of epochal evolution which tends to generate fairly regular intervals of time, and cannot explain constant diversity, between selective sweeps [10]. We propose that future work on the dynamics of the influenza virus should attempt to replicate the detailed patterns of population turnover and antigenic evolution, rather than focusing on the larger scale assessment of general tree shape. Investigation of the effects of space and the shape/topology of the genotype-phenotype map should complement these efforts.

## Methods

### Sequence data

To examine broad genealogical patterns influenza (Figure 1), sequences belonging to the hemagglutinin (HA) gene were downloaded from the Influenza Virus Resource of GenBank [41]. Only non-lab strains possessing at least 900 bases and recorded years of collection were used. To obtain a more even distribution of samples over time, we further sampled at most 10 sequences per year from 1968 to 2008, leaving a total of 363 sequences for analysis. Similarly, to ascertain genealogical patterns in measles (Figure 1), we obtained measles virus sequences belonging to the hemagglutinin (H) gene from GenBank [42]. Sequences had to be at least 1800 bases and possess dates of collection. Vaccine strains and strains associated with the persistent disease manifestation subacute sclerosing panencephalitis (SSPE) were removed from the analysis. These procedures resulted in 190 sequences for analysis.

For the more detailed analysis of influenza (Figure 8, Table 2), sequences belonging to the hemagglutinin (HA) gene were downloaded from the Influenza Virus Resource of GenBank [41]. Only non-lab strains of at least 900 bases with fully specified dates (day, month, year) and countries of origin were used. We restricted our analysis to sequences dated from 2000 to 2008. We categorized the resulting samples into 7 geographic regions (China, Europe, Japan, Oceania, South America, Southeast Asia and the USA). This resulted in 3399 samples. Due to data gathering procedures, these data are biased towards recent samples and towards samples from the USA and New Zealand. Thus, we sought a more equitable sampling distribution by further sampling at most 5 sequences at random for each month in each region. This procedures smoothed out differences in temporal and spatial sampling patterns, resulting in a final tally of 1270 sequences. To control for spatial effects, we replicated the analysis for sequences restricted to China and Southeast Asia. Here, we sampled at most 15 sequences per month in each region. This procedure left 624 sequences for analysis.

In all cases, sequences were aligned using MUSCLE v3.7 under default parameters [43].

### Population genetic simulations

An evolving population of sequences was simulated according to a haploid Wright-Fisher model with discrete generations. A Moran model may be more analogous to an evolving pathogen population. However, it has been shown that the Wright-Fisher and Moran models are completely equivalent with a 2× rescaling of generations [16]. Each sequence was 2000 bases (chosen as 'A', 'C', 'G' or 'T') in length. We used a Jukes-Cantor mutation model with a mutation rate of 10^-5 ^per site and per generation. In each subsequent generation, the population was reconstituted by sampling sequences with replacement proportional to their frequency multiplied by their fitness. Mutations affected fitness in a multiplicative fashion (additive on a log-scale). The proportion of mutations that had selective impact and the strength of this impact were varied between simulations. All simulations were run for 5 × 10^4 ^generations before logging any samples. Sequences were sampled at random time points after this period of 'burn-in.' Simulation source code and executables are available from the authors upon request.

### Coalescent inference

Evolutionary dynamics were estimated using a Bayesian coalescent model that describes the genealogical relationships among observed sequences. This model is evaluated using Markov chain Monte Carlo (MCMC). MCMC explores the parameter-space through a random walk, converging on the posterior distribution of the model parameters. Evolutionary parameters and genealogical trees were estimated using the MCMC techniques implemented in the coalescent inference program BEAST v1.6.0 [25]. Here, trees are constructed following a coalescent process, which imposes a prior on the branch lengths of the tree. We used the GTR model of nucleotide evolution to parameterize the mutational process, with the evolutionary rate across sites held constant. Each relative rate parameter in the GTR model was assumed to follow a non-informative Jeffreys prior. The overall mutation rate *μ *was assumed to follow a uniform prior distribution. A piece-wise skyline population model with 10 discrete sizes model was assumed [44]. The same model was used for all three simulated datasets and for both influenza datasets. Each population size parameter was assumed to follow a Jeffreys prior. Each MCMC chain was run for 40 million steps with the first 20 million steps discarded as 'burn-in.' Samples were logged every 10,000 steps, providing a total of 2000 samples for each analysis.

Genealogical trees sampled by BEAST were analyzed using the software package PACT v0.9.3 (source code available from the author's website at http://www.trevorbedford.com/pact). Posterior 'skyline plots' of descriptive statistics TMRCA and diversity were produced by taking a series of temporal slices of lineages. At each point in time, this slice produces a subtree consisting of only ancestral lineages. This procedures transforms a genealogy with temporal spacing of tips into a genealogy with tips at only a single point of time. For each subtree, we then calculated the depth of the common ancestor (TMRCA) and the mean branch distance between pairs of tips (diversity). Our calculation of the *N_e_τ *statistic differs from the calculation of the 'classic skyline plot' [23] and also the 'generalized skyline plot' [24]. In our implementation, we take a fixed windows of time and calculate the opportunity for coalescence in this window and divide this opportunity by number of coalescent events observed to yield *N_e_τ*. To arrive at the opportunity for coalescence we find coalescent intervals [16] and calculate:

where *n *is the number of concurrent lineages, *t *is the length of the interval and *i *represents the discrete set of intervals. If *n *equals 0 or 1, then the opportunity in this interval is 0. The sliding window width was chosen to optimize temporal resolution while leaving enough data in each window to keep the credible interval sufficiently narrow. We chose windows of 50 generations for the simulation studies and 0.25 years for the influenza analysis to accomplish this. Using smaller windows results in a 'bumpier' time-series with wider credible intervals and using larger windows results in a smoother timeseries with narrower credible intervals. Our basic results are robust to choice in window size. Each calculation was run on all 2000 sampled genealogies and from the distribution of results, means and 95% credible intervals were obtained.

Genealogical trees were drawn using the program PACT. Each tree shown represents the sample with the highest posterior probability in the MCMC analysis.

## Authors' contributions

TB, SC and MP conceived of the project. TB conducted the simulation experiments, gathered the biological data and performed statistical analyses. TB, SC and MP analyzed the results and wrote the paper.
